# Negatively Charged Porous Thin Film from ABA Triblock Copolymer Assembly

**DOI:** 10.3390/polym10070733

**Published:** 2018-07-03

**Authors:** Sabrina Nehache, Mona Semsarilar, André Deratani, Damien Quemener

**Affiliations:** Institut Européen des Membranes—IEM, Université de Montpellier, CNRS, ENSCM, Place Eugène Bataillon, 34095 Montpellier CEDEX 05, France; sabrina.nehache@hotmail.fr (S.N.); andre.deratani@univ-montp2.fr (A.D.)

**Keywords:** block copolymer, self-assembly, porous film, membrane

## Abstract

The preparation of well-arranged nano-porous thin films from an ABA triblock copolymer of polystyrene-*block*-poly(sodium 4-styrenesulfonate)-*block*-polystyrene (PS-PNaSS-PS) is reported. This copolymer was self-assembled in a *N*,*N*-dimethylformamide (DMF)/water mixture and the resulting micellar solution was used to prepare thin films via the compact packing of the flower-like micelles using spin coating method. The films were characterized by several microscopy techniques such as TEM, AFM, and SEM. Permeation test was performed to highlight the interconnected porous nature of the polymeric network obtained. Under applied water pressure, the micellar morphology was altered and a partial fusion of the micelles was observed that resulted in a change in the water permeability. Such hydrophilic nanoporous thin films with negatively charged interface could find applications in membrane filtration.

## 1. Introduction

Materials made of block copolymer assembly facilitate the construction of complex regular structures by using simple process. In fact, copolymers can micro-phase separate into well-defined structures in solution, in bulk, and in thin films, thus creating a library of structures and assemblies [1,2,3,4,5,6,7,8,9,10,11,12,13]. These well-arranged structures have been used in various nanotechnological fields from drug delivery to solar cells and membranes [2,14,15,16]. The interest of having block copolymer in emerging materials is that the morphologies could be easily tuned according to molecular weight, copolymer structures, and solvent composition [17]. In the recent years, there has been a huge interest in preparation of nanoporous material from block copolymers [18,19,20,21]. Porous materials have been made via phase inversion process [22], etching [23], and photo-degradation [20]. The narrow pore size distribution and ability to tune the pore size allowed by the use of block copolymers is attractive for the formation of membrane materials with controlled selectivity for applications such as ultrafiltration or drug delivery [21,24,25,26]. Another important emerging approach is the self-assembly and non-solvent-induced phase separation (SNIPS) of block copolymers [27,28]. Polystyrene-*b*-poly(4-vinylpyridine) (PS-*b*-P4VP) is by far the most explored block copolymers in the SNIPS process [21]. If the direct use of charged block copolymers remains largely unexplored for the formation of nanoporous material, a substantial number of articles describe the preparation the porous films followed by their subsequent conversion into a charge polymer. Positively charged SNIPS membranes are classically made from the quaternization of the hydrophilic part under acid conditions, as for example for P4VP [29] or poly(dimethylaminoethyl methacrylate) (PDMAEMA) blocks [24]. Less works have been reported on SNIPS polyanionic membranes. *pH*-sensitive membranes could be made from poly(acrylic acid)-based block copolymers [30], or after the post-modification of P4VP-based membranes into P4VP N-oxide via a selective oxidation step [31].

The development of hydrophilic materials is among the requisites to the growing of the membrane technologies in order to control the operational costs (material ageing, cleaning, energy consumption, etc.). In that context, linear polyelectrolytes have received growing interest as they are essential for water-based applications such as sensors [26], environmental applications [32], transport layers for fuel cells [33], water purification [34], lubrication [35], protein adsorption [36], or for complexation of DNA to prepare gene transfection agents [37]. Polyelectrolytes can be weak or strong and can display ionization behaviour based on the pH. As strong acids are pH-independent, their integration in a material can prevent its swelling even at low *pH*. Poly(sodium 4-styrenesulfonate) (PNaSS) is especially known for its ability to reduce the amount of potassium in the body [38], for its utilisation as ion exchange material [39], ion transport [40,41] and in proton conduction in fuel cell membranes [42].

Herein, the preparation of well-arranged nano-porous thin films from an ABA triblock copolymer of polystyrene-*block*-poly(sodium 4-styrenesulfonate)-*block*-polystyrene (PS-PNaSS-PS) is reported. The morphology of the copolymer in *N*,*N*-dimethylformamide (DMF)/water solvent mixture was studied and thin films were prepared by spin coating. The resulting materials were characterized using AFM, TEM, and SEM techniques. Finally, the pure water transport across the nano-porous thin film was studied by applying a pressure gradient across the films.

## 2. Materials and Methods

### 2.1. Materials

All reagents were purchased from Merck (Darmstadt, Germany) and were used as received unless otherwise stated. Azobisisobutyronitrile (AIBN) was purchased from Merck (Darmstadt, Germany) and was recrystallized from methanol. Styrene (S) was passed through basic alumina prior to use. *N*,*N*-dimethylformamide (DMF) and tetrahydrofuran (THF) were purchased from Merck (Darmstadt, Germany). Deuterated chloroform (CDCl_3_) and dimethylsulfoxide ((CD_3_)_2_SO) were purchased from Eurisotop (Saint-Aubin, France).

*Synthesis of S,S′-bis-(α,α′-disubstituted-α″-acetic acid)-trithiocarbonates (DTTC).* This compound was synthesized following the method described by Lai et al. [43]. Under inert atmosphere (N_2_), 120.0 mL of mineral spirits was mixed with carbon disulfide (27.4 g, 0.36 mol), chloroform (107.5 g, 0.90 mol), acetone (52.3 g, 0.90 mol), and tetrabutylammonium bisulfate (2.41 g, 7.10 mmol) in a 1.0 L three neck round bottom flask. Sodium hydroxide (50%) (201.6 g, 2.52 mol) was added drop wise over 90 min keeping the temperature under 25 °C. The reaction was stirred overnight. The yellowish solid was then dissolved in 900 mL of distilled water, followed by 120 mL of concentrated HCl. The mixture was stirred for 30 min in a stream of nitrogen. The solid was collected and washed thoroughly with distilled water. The yellow product was recrystallized in acetone. ^13^C NMR (MeOD, δ (ppm)): 25.76, 57.25, 176.26, 220.50.

*Synthesis of poly(styrene) macromolecular chain transfer agent (PS).* In a typical experiment, a round-bottomed flask was charged with styrene (S) (20 g, 192 mmol), DTTC (0.13 g, 0.48 mmol, dissolved in 2.0 mL of DMF), and AIBN (42 mg, 0.24 mmol). The sealed reaction vessel was purged with nitrogen and placed in a preheated oil bath at 70 °C for 20 h. The resulting PS macro-CTA (50% conversion; *M*_n_ = 20,100 g·mol^−1^, *M*_w_ = 22,500 g·mol^−1^, *Ð* = 1.11) was purified twice by precipitation into excess methanol and dried under vacuum overnight. The conversion was calculated by ^1^H NMR spectroscopy by comparing the integrated aromatic proton signals due to the PS groups at 6.4–7.6 ppm to those due to the monomer (S) at 5.3–5.8 ppm.

*Synthesis of polystyrene-block-poly(sodium 4-styrenesulfonate)-block-polystyrene triblock copolymer (PS_10K_-PNaSS_10K_-PS_10K_).* In a typical experiment, a round-bottomed flask was charged with PS_20K_ (6.15 g, 0.306 mmol, dissolved in 8.0 mL of DMF), AIBN (12.5 mg, 0.073 mmol), and sodium 4-styrenesulfonate (NaSS) (3.06 g, 14.8 mmol dissolved in 50.0 mL DMF). The sealed reaction vessel was purged with nitrogen and placed in a preheated oil bath at 70 °C for 22 h. The resulting polymer PS_10K_-PNaSS_10K_-PS_10K_ (100% conversion, target PNaSS molecular weight was 10,000 g·mol^−1^) was purified by dialysis against water and isolated by freeze-drying.

*Preparation of PS-PNaSS-PS triblock copolymer micelles.* An amount of 0.10 g of the triblock copolymer (PS_10K_-PNaSS_10K_-PS_10K_) was dissolved in DMF (0.50 g) and left stirring overnight. Then 0.07 g of ultrapure water was added gradually at a rate of 0.01 g·min^−1^ to obtain the micelles. The solution was then left stirring overnight before the thin film preparation.

*Preparation of PS-PNaSS-PS triblock copolymer thin film.* Thin films were produced from the as-prepared micelle solution of triblock copolymer PS_10K_-PNaSS_10K_-PS_10K_ in DMF/ultrapure water. The solution was spin coated onto a clean silicon wafer at 1000 rpm for 240 s with a speed ramp of 100 rpm·s^−1^ under a dry argon atmosphere. For SEM analysis, the thin films were detached from the silicon wafers by floating in ultrapure water. The detached films were then supported onto non-woven polyester fabric and dried at room temperature. The AFM analyses were performed directly on the silicon wafer or on the Nylon membrane.

### 2.2. Analytical Techniques

*^1^H NMR spectroscopy.* NMR spectra were acquired in either CDCl_3_ or a mixture of CDCl_3_ and (CD_3_)_2_SO using a Bruker 300 MHz spectrometer (Billerica, MA, USA). All chemical shifts are reported in ppm (δ).

*Size exclusion chromatography (SEC).* Molecular weight distributions were assessed by size exclusion chromatography (SEC, Malvern Panalytical, Orsay, France)) using THF or DMF as eluent. PS block SEC analyses were performed with THF as eluent, at a flow rate of 1.0 mL/min on a Polymer Laboratories PL-GPC 50 instrument using two PL mixed C 5.0 µm columns at 35 °C and a refractive index detector. Calibration was done using Varian polystyrene narrow standards. A smaller molecular weight triblock copolymer PS_5K_-PNaSS_5K_-PS_5K_ was synthesized to check the polydispersity index as the PS_10K_-PNaSS_10K_-PS_10K_ was not soluble in either DMF or THF alone. For PS_5K_-PNaSS_5K_-PS_5K_, SEC analyses were performed with DMF as eluent, since the polymer was insoluble in THF and water. These analyses were performed on a Spectra-Physics apparatus equipped with a set of two PL gel 5.0 µm C mixed-columns from Polymer Laboratories at a flow rate of 1.0 mL·min^−1^. The calibration curve was established using PMMA standards from Polymer Laboratories ranging from 600 to 128,000 g·mol^−1^. DMF was of HPLC grade and filtered at 0.20 µm prior to use as SEC eluent.

*Spin Coating.* Polymer thin films were prepared using a SPS Spin 150 Spin coater (Vourles, France) at 1000 revolutions per minute (rpm) for 240 s with a speed ramp of 100 rpm·s^−1^ under a dry argon atmosphere.

*Scanning Electron Microscopy (SEM).* SEM analyses were conducted using a Hitachi S-4500 (Tokyo, Japan) instrument operating at spatial resolution of 1.50 nm at 15 kV energy. The samples were dried and coated with an ultrathin layer of electrically conducting platinum deposited by high-vacuum evaporation.

*Atomic force microscopy (AFM)*. AFM images were obtained with a Pico SPM II (Tempe, CA, USA) provided by Molecular Imaging. The imagery was controlled by the PicoView 1.10 software (Molecular Imaging, Tempe, CA, USA). The experiments were all carried out in tapping mode. The type of tips used were PPS-FMR purchased from Nanosensors (Neuchatel, Switzerland) with a frequency resonance between 45 and 115 kHz and a force constant between 0.5 and 9.5 N/m. Gwyddion 2.25 software (Gwyddion, Brno, Czech Republic) was used to treat the images. The 2-Dimensional Fast Fourier Transform was performed with the 2D FFT function available on Image J software (Image J, Bethesda, MD, USA).

*Transmission electron microscopy (TEM).* TEM studies were conducted using a JEOL 1200 EXII (Croissy-sur-Seine, France) instrument operating at 120 kV equipped with a numerical camera. To prepare TEM samples, 5.0 μL of a dilute copolymer solution was placed onto a carbon-coated copper grid, stained using an aqueous solution of ammonium molybdate 99.98%, and then dried under ambient conditions.

*Water Contact angle (WCA).* The WCA was measured using a monochrome camera B-CAM-21-BW (CCCIR) and a Led R60 lamp purchased from CONRAD (Sequedin, France). For each sample, 10.0 µL of ultra-pure water was deposited on a polymer coated silicon wafer using a needle. The images were recorded by One Touch Graber software and treated using Matlab software (MathWorks, Natick, MA, USA).

*Zeta potential measurement.* The surface charge (zeta potential) of the membrane was measured using SurPASS electrokinetic analyzer (Anton Paar, GmbH, Graz, Austria) based on the streaming potential method. Washed membrane samples were mounted in an adjustable gap cell and soaked in 1 mM KCl. The cell height was fixed at 100 mm. The electrolyte solution was circulated in the cell between two pieces of membrane. The zeta potential was calculated using the Helmholtz–Smoluchowski equation from the measured streaming current as a function of *pH*.

*Water permeation experiments.* The triblock copolymer solution was spin coated on a commercial Nylon membrane (Fiorini filter, ref. 0462A0023, with average pore size of 0.2 µm). The coated membrane was mounted in a dead-end filtration cell (Millipore, 10 mL) and subjected to water pressure between 0 and 4.0 bars. Before recording the water flux, the coated film was conditioned/compressed for 2 h at 4.0 bars to reach an equilibrium state.

## 3. Results

### 3.1. Synthesis of PS_10K_-PNaSS_10K_-PS_10K_

The RAFT synthesis of the triblock copolymer PS_10K_-PNaSS_10K_-PS_10K_ was performed in two steps as reported previously [44,45]. Polystyrene (PS) was polymerized using DTTC as the chain transfer agent (CTA) and AIBN as the radical initiator (CTA/initiator molar ratio = 1:0.5) in bulk. A small amount of DMF solvent was used to ensure complete dissolution of the CTA. The reaction was stopped after 20 h (50% conversion, calculated by ^1^H NMR) targeting a *M*_n_ of 20,000 g·mol^−1^. The resulting PS macro-CTA was purified by precipitation in methanol. The SEC analysis in THF suggested a *Ð* of 1.1 and *M*_n_ of 20,000 g·mol^−1^. The second step was performed under similar RAFT polymerization conditions. The synthesized near monodisperse PS macro-CTA was dissolved in DMF along with sodium 4-styrenesulfonate (NaSS) and AIBN. The macro-CTA/initiator molar ratio was fixed at 1:0.25, and NaSS polymerization was conducted at 70 °C for 22 h (100% conversion, target *M*_n_ = 10,000 g·mol^−1^). The resulting PS_10K_-PNaSS_10K_-PS_10K_ was purified by dialysis against water. As the final synthesized triblock copolymer was not soluble in common SEC eluents (THF, DMF, water), a lower molecular weight (PS_5K_-PNaSS_5K_-PS_5K_) was synthesized to check the control over the polymerization. The narrow *Ð* of 1.17 (determined by SEC using DMF as eluent) suggests a sufficient control over the polymerization [44]. A Diffusion Ordered Spectroscopy (DOSY) NMR analysis was also performed in DMSO-*d*_6_ to confirm the blocking (see reference [44] for the characterization of the copolymers).

### 3.2. Block Copolymer Micelle Preparation

To achieve the self-assembly, the copolymer, PS_10K_-PNaSS_10K_-PS_10K_, was dissolved in DMF (as a relatively good solvent for both PS and PNaSS) and then under vigorous stirring, water (only selective for PNaSS) was added dropwise to reach the desired DMF/water ratio (Table S1). A yellowish lightly turbid solution is observed when the copolymer is initially dissolved in DMF. Upon the addition of water, flower-like micelles are formed with hydrophobic PS chains in the core and hydrophilic PNaSS corona resulting in a milky colloidally stable solution. A low contact angle value (10°, see Table S1) of a film made from the spin coated solution, confirmed the formation of the flower-like micelles. The hydrophilicity of the surface is in fact due to the presence of the PNaSS chains forming the micelles corona at the film surface.

**Figure 1:**
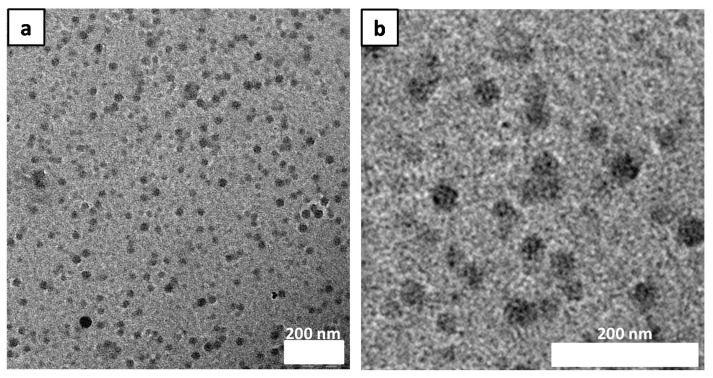
(**a**) TEM image of PS_10K_-PNaSS_10K_-PS_10K_ diluted in DMF/water, stained and dried under ambient conditions (**b**) Enlarged image of (**a**).

To observe the morphology of the micelles, the copolymer solution was diluted with the same DMF/water ratio used to make the original solution. The solution was dried on a TEM grid and stained using ammonium molybdate solution (Figure 1a,b). Figure 1 shows small well-defined spherical micelles with diameter of 30 nm.

### 3.3. Film Formation and AFM Observation

**Figure 2:**
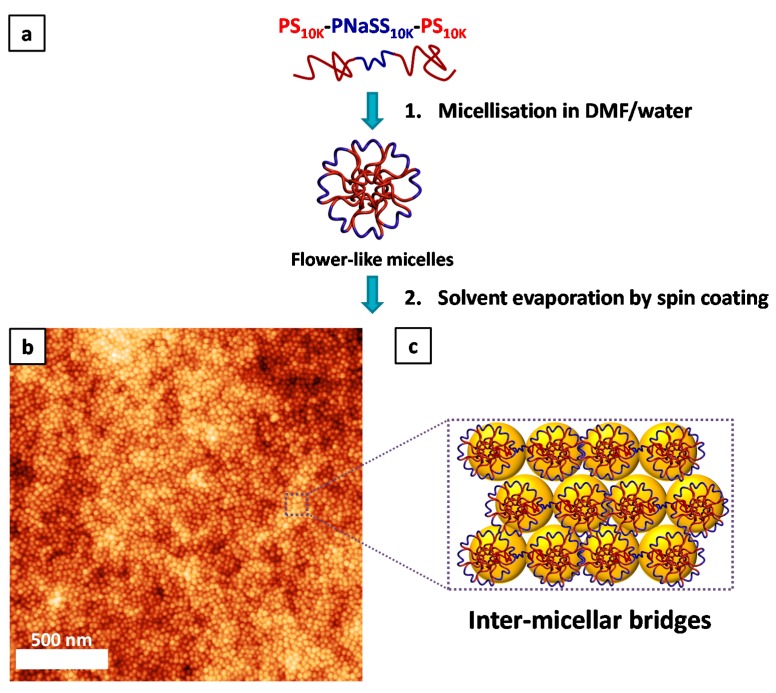
(**a**) Micelle and thin film formation (**b**) AFM image of the film formed using the copolymer micelle solution (**c**) Representation of the inter-micellar bridges between the flower-like micelles maintaining the film structure.

Figure 2 illustrates the process used to make the thin films from the copolymer solution. The first step (Figure 2(a1)) consists of making the flower-like micelles in DMF/water mixture, as previously explained. This solution is then spin-coated onto a silicon wafer (Figure 2(a2)). The evaporation of the solvent results in the assembly of the spherical micelles and the free volume left between them impart the porosity to the film. The structure of the thin film prepared via spin coating from the block copolymer solution (Table S1) was analyzed by atomic force microscopy (AFM) (Figure 2b). The AFM phase image of this film shows no phase contrast (Figure S1b), which suggests that only the PNaSS blocks are present on the surface. Measurements in the cross-sectional profile of AFM topography images reveal that the average micelle size in diameter is 30 nm (Figure S2) (average value found for the width difference between peaks). This value is in agreement with the average micelle diameter found by TEM measurement and was confirmed by a Fast Fourier Transform (FFT) analysis. A FFT was performed on Figure 2b to determine the characteristic length of the porous structure (Figure S3). A characteristic length *L_c_* of 31 nm was calculated according to Equation (S1) that corresponds to size of one the spherical micelles as illustrated in; Figure S4. The compact micelle assembly observed in Figure 2b is in fact engendered by bridging links between the flower-like micelles (Figure 2c) as demonstrated before [46].

### 3.4. SEM and Film Thickness

**Figure 3:**
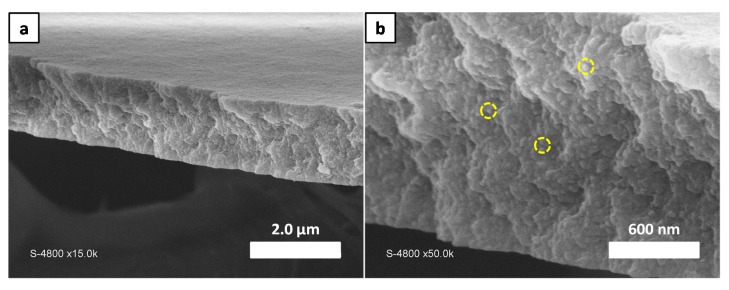
SEM images of the detached thin film after spin coating (**a**) Film cross section (**b**) Enlarged image of (**a**). Yellow doted circles highlight the presence of the spherical micelles inside the film.

To determine thoroughly the structure of the film formed by this sample, its thickness was measured using SEM microscopy (Figure 3). To do so, the film was first detached from the silicon wafer via immersion in ultrapure water and then dried for analyses. Figure 3 displays cross section of this film. These images confirm that the film formed from micelle assembly is mechanically strong enough to maintain its integrity even after detachment from the silicon wafer. The thickness of the obtained film was approximately 1.5 µm. The enlarged cross section image in Figure 3b shows a homogeneous compact assembly of spherical micelles all across the film thickness. It can thus be assumed that an interconnected porous network is formed via the interspace between micelles from the top to the bottom of the thin film.

### 3.5. Water Permeation Experiments

The nanoporous thin film formed was then used as a synthetic water filtration membrane. The porosity of the top surface of the film was evaluated by image treatment and was found to be around 44% (Figure S5, Equation (S2)). Since the pore size is inferior to 5 nm (AFM observations) the film could potentially be used to filtrate sugars, synthetic dyes, pesticide, and herbicide [47]. 

**Figure 4:**
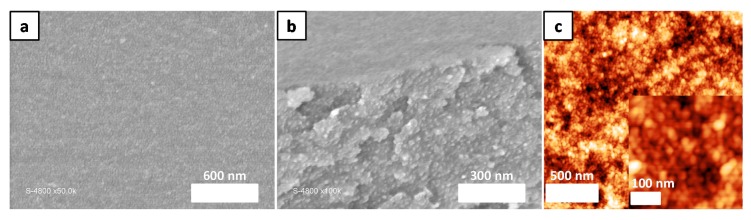
SEM images of the top surface (**a**) and cross section (**b**) of the support membrane coated with micellar solution of PS_10K_-PNaSS_10K_-PS_10K_ in DMF/water mixture. (**c**) AFM image showing the spherical micelle assembly.

As seen in Figure 3, the film was robust enough to keep its structure after the detachment drying process. However, its mechanical properties were not sufficient to tolerate water filtration under pressure. To perform the filtration test, the copolymer solution (Table S1) was directly spin coated on a commercially available Nylon membrane (with pore sizes of 200 nm). As the commercial membrane has pores almost 40 times bigger than the copolymer film, the filtration results should not be prevaricated. The homogeneous coating of the copolymer was first confirmed by SEM analyses (Figure 4a) before the filtration test. By comparing the SEM images of the virgin Nylon membrane (Figure S6) with the coated membrane (Figure 4a,b), homogeneous deposition of the copolymer micelles solution was confirmed. To make sure the spherical micelle shape and assembly were maintained after the coating on the Nylon membrane, AFM analysis was also performed (Figure 4c).

**Figure 5:**
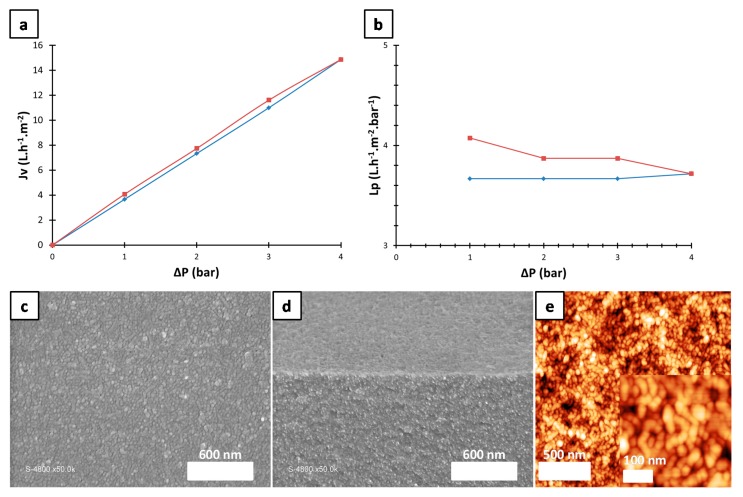
(**a**) Flux of the coated membrane determined by water filtration at different pressures. (**b**) Permeability of the coated membrane determined by Darcy’s law at different pressures (blue indicates increasing pressure and red indicates the decreasing pressure). (**c**,**d**) SEM image of the top surface (**c**) and cross section (**d**). (**e**) AFM image of the deformed micellar assembly after water permeation.

To perform water permeation, the coated membrane was mounted in a filtration cell. The water flux and the permeability of the membrane were measured for pressures up to 4.0 bars. The results are shown in Figure 5a,b. First, the pressure was increased progressively (1.0 bar every two hours, Figure 5a,b blue trace). After reaching 4.0 bars, the pressure was gradually decreased by 1.0 bar at two hour intervals (Figure 5a,b red trace). The flux ($J_{v}$) and the permeability ($L_{p}$) of the membrane were calculated according to Darcy’s laws (Equations (1) and (2)):

(1) $$J_{v}=\frac{V_{p}}{tS}$$

(2) $$L_{p}=\frac{J_{v}}{\Delta P}$$

where $V_{p}$, t, S, ΔP correspond to water volume going through the membrane (L), time (h), surface of the membrane (m^2^), and water pressure (bars), respectively. The water flux first increased and then decreased linearly with the increasing and decreasing pressure. The permeability stayed constant between 1.0 to 4.0 bars (4 L·h^−1^·m^−2^·bar^−1^). As it can be seen in Figure 5a,b, the blue and the red traces are almost superimposed however an offset is observed. In fact, $J_{v}$ and so $L_{p}$, corresponding to pressure decrease (red), are slightly higher than those for the pressure increase (blue). To understand this shift of the flux and permeability, the membrane was dried and analyzed by SEM and AFM. Figure 5c–e displays the surface and the cross section of the membrane after filtration. Some structural changes (spheres to worm-like micelles) are observed after carrying out the water filtration under pressure. The AFM image (Figure 5e) also confirms that, under water pressure, the spherical particles deform and some of them fuse to form worm-like micelles. This change in the morphology leads to changes in the porosity of the membrane. The cross-sectional AFM profile shown in Figure 5e shows that the micelle size is also changed after the filtration test (from 30 nm to 45 nm) (Figure S7). This increase in size suggests that a partial fusion of the micelles has occurred thus changing the porosity and the pore size. This could also explain the slight shift observed in one cycle of filtration (between the blue and the red traces of flux and permeability). Similar phenomenon has been reported previously by our team [46] where a structural deformation of the block copolymer micelles was controlled using the water pressure.

It could be noted that the membrane has a pore size corresponding to a membrane used for nanofiltration but demonstrate a permeability closer to reverse osmosis membrane [47]. However, a high tortuosity of the pores combined to a high film thickness dramatically affects the permeability. Therefore, a commercial application as a filtration membrane would require further optimization.

Finally, as the PNaSS is known to be charged in water, zeta potential measurement was performed (Figures S8 and S9) at a pH range varying from 5 to 8, to evaluate the surface charge of the film. First, the zeta potential of the virgin Nylon membrane was measured (Figure S8) to evolve from −2.7 mV (pH = 5.3) to −29.6 mV (pH = 7.5). As expected, when surface of the Nylon membrane was covered with the copolymer micelle bearing SO_3_^−^ groups on its surface, the zeta potential value remained constant independently of the pH changes (Figure S9) since sulfonic acid group is a strong acid with a *pKa* = −3. The zeta potential varied from −31.40 mV (*pH* = 5.3) to −33.53 mV (*pH* = 7.5).

Therefore, as the surface of the membrane is hydrophilic and charged (shell of PNaSS, *CA* = 10°), this film could be considered for metal ion filtration [48] since the sulfonate group is able to form complexes with metals, or as an exchange membrane due to the charge of PNaSS in water [49,50,51,52].

## 4. Conclusions

To summarize, an amphiphilic triblock copolymer of PS_10K_-PNaSS_10K_-PS_10K_ was synthesized via RAFT controlled polymerization. This copolymer was then self-assembled in a DMF/water solvent mixture and the resulting micellar solution was used to prepare thin films via the compact packing of the flower-like micelles using the spin coating method. These films were characterized by several microscopy techniques such as TEM, AFM, and SEM. The porous material was finally supported on a commercial Nylon membrane to perform preliminary water permeation test. Under water pressure, the material underwent some structural changes (individual to fused micelles) leading to a slight change in the pure water permeability. This work strengthens the importance of controlling the solvent selectivity when preparing a block copolymer film. In a medium selective enough, nano-porous materials can be obtained with a pore size mainly controlled by the size of the micellar building blocks. These micellar block copolymer materials could be used in several applications such as for example ultrafiltration for water treatment, selective separation of solutes for pharmaceutical and food industry, drug delivery, and medical filtration needs such as dialysis and data storage.

## Supplementary Materials

The following are available online at http://www.mdpi.com/2073-4360/10/7/733/s1, Table S1: PS_10K_-PNaSS_10K_-PS_10K_ solution composition and film characteristic; Figure S1: AFM images of the film prepared with PS_10K_-PNaSS_10K_-PS_10K_ copolymer micelle solution (a) Amplitude image (b) Phase image; Figure S2: AFM cross section profile (a) Zoom of the AFM topography image of PS_10K_-PNaSS_10K_-PS_10K_ copolymer solution (b) Cross-sectional profile of the topography along the line shown in image (a) (c) 3D view of the AFM topography image; Figure S3: (a) Topography AFM image of PS_10K_-PNaSS_10K_-PS_10K_ after spin coating (b) FFT of image (a) (c) Mathematic treatment of the FFT, obtained by circular mean, of topography image (a); Figure S4: Illustration of the *L_c_* on the AFM topography image. Lc represents the size of one spherical micelle; Figure S5: (a) SEM image of PS_10K_-PNaSS_10K_-PS_10K_ after drying by spin coating and detachment from the wafer by immersion in water (b) image (a) binarized using Matlab software (c) Diagram of Normalized intensity of the pixels versus pixel obtained by the data treatment on image (b) using Matlab software; Figure S6: SEM images of the virgin Nylon membrane used as support for the coating of PS_10K_-PNaSS_10K_-PS_10K_ (a) Top surface (b) Zoom of the top surface (c) Bottom surface (d) Cross-section of the uncoated membrane; Figure S7: AFM 3D view and profile of the coated membrane after filtration cycles under pressure; **Figure S8**: Zeta potential curve versus pH for the uncoated commercial Nylon membrane; **Figure S9**: Zeta potential curve versus pH for the uncoated commercial Nylon membrane.
