# Supplementary material for: Negatively Charged Porous Thin Film from ABA Triblock Copolymer Assembly
Source: Polymers (Basel). 2018 Jul 3;10(7):733. doi: 10.3390/polym10070733 (PMC6403756; doi:10.3390/polym10070733)
Supplement: Supplementary file 1 [file polymers-10-00733-s001.pdf]

SUPPORTING INFORMATION

Negatively charged porous thin film from ABA triblock copolymer assembly

Sabrina Nehache [a] , Mona Semsarilar [a,]*, André Deratani [a], Damien Quemener [a,]*

[a] Institut Européen des Membranes – IEM, Univ Montpellier, CNRS, ENSCM, Place Eugène Bataillon, 34095 Montpellier Cedex 05, France.

Table S1. PS_{10K}-$PNaSS_{10K}$-PS_{10K} solution composition and film characteristic.

Samples	Polymer (g)	DMF (g)	Water (g)	Contact Angle (°)	Particle Shell	Zeta potential of the film[a] (mV)
**PS_{10K}-$PNaSS_{10K}$-PS_{10K}**	0.10	0.50	0.07	10	PNaSS	-32.25

[a] Average value based on 3 measurements at pH 7.

AFM supplementary information

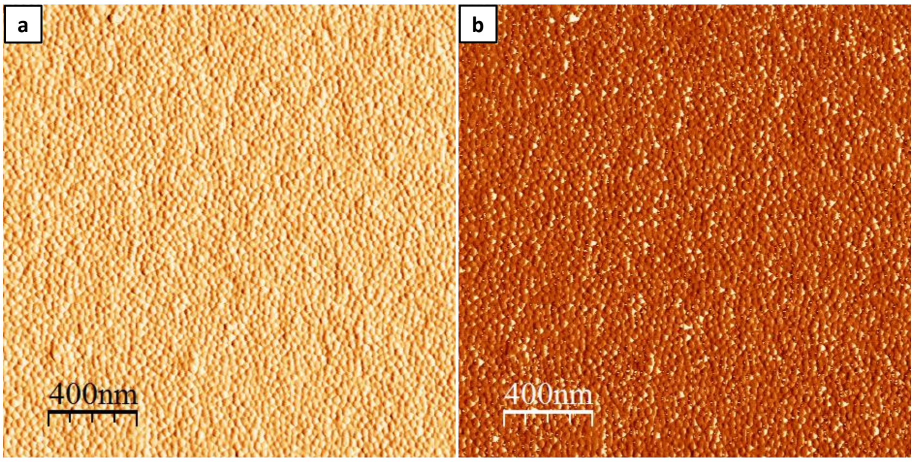

Figure S1. AFM images of the film prepared with PS_{10K}-$PNaSS_{10K}$-PS_{10K} copolymer micelle solution (a) Amplitude image (b) Phase image.

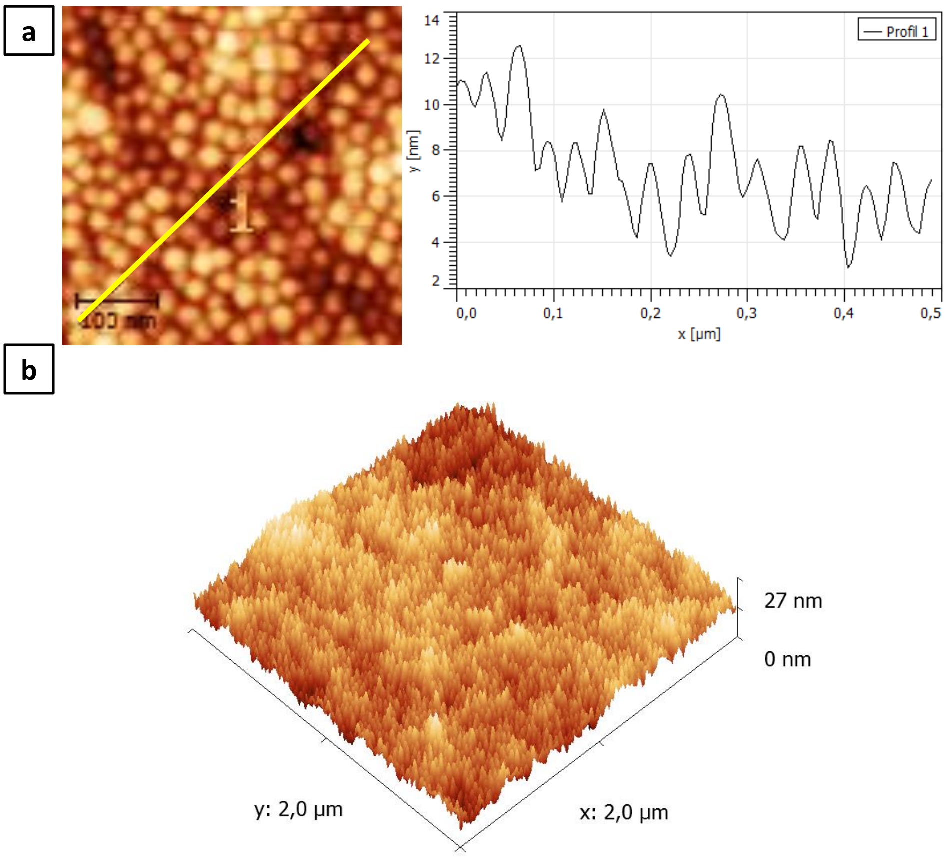

Figure S2. AFM cross section profile (a) Zoom of the AFM topography image of PS_{10K}-$PNaSS_{10K}$-PS_{10K} copolymer solution (b) Cross-sectional profile of the topography along the line shown in image (a) (c) 3D view of the AFM topography image.

Fast Fourier Transform (FFT)

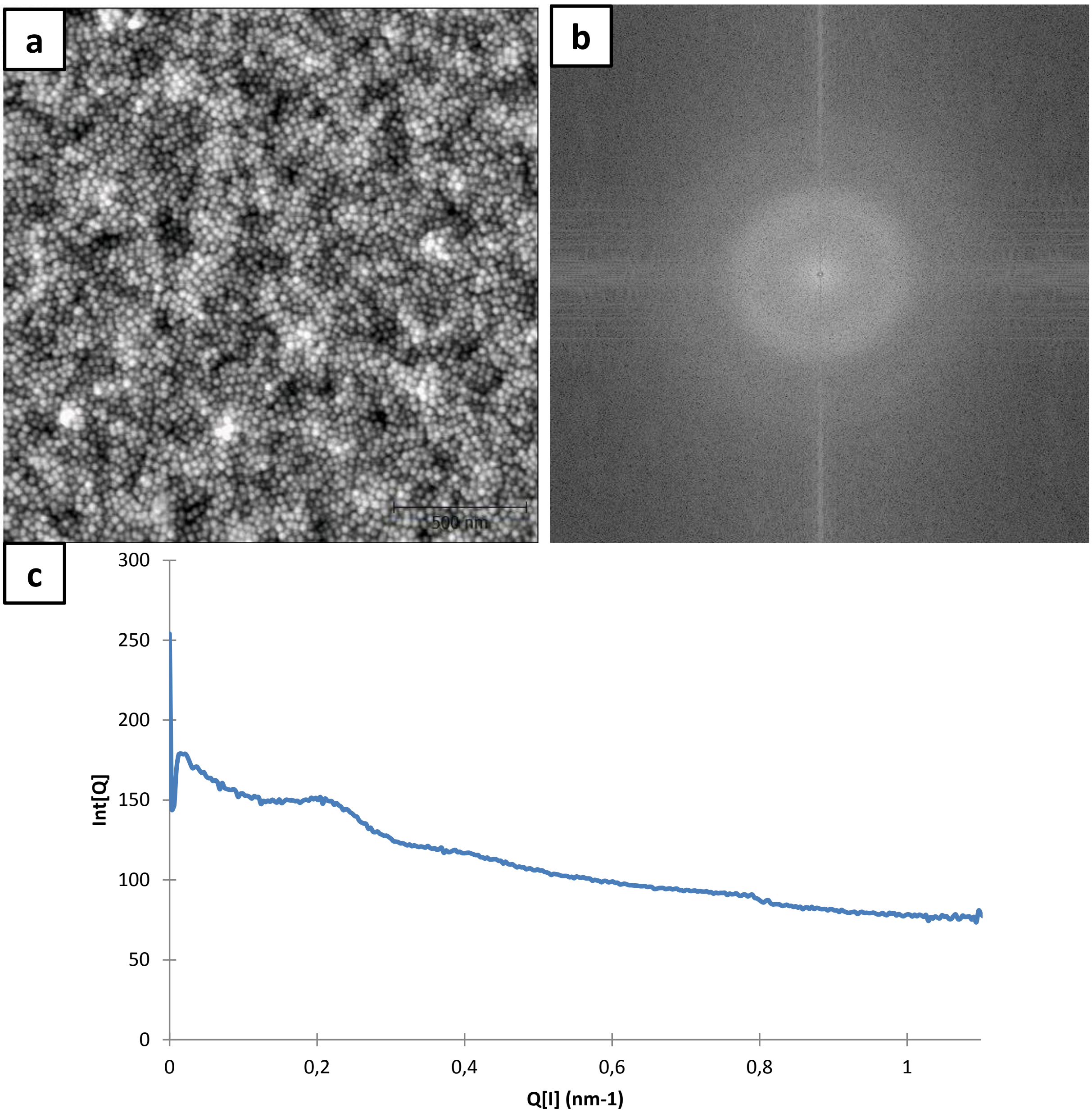

Figure S3. (a) Topography AFM image of PS_{10K}-$PNaSS_{10K}$-PS_{10K} after spin coating (b) FFT of image (a) (c) Mathematic treatment of the FFT, obtained by circular mean, of topography image (a).

$$Q_c = \mathbf{0{,}2014\ nm^{-1}}$$

The characteristic length $\boldsymbol{L_c}$ was calculated according **Equation S1**:

$$L_c = \frac{2\pi}{Q_c} = 31.19\ nm \qquad \text{(S1)}$$

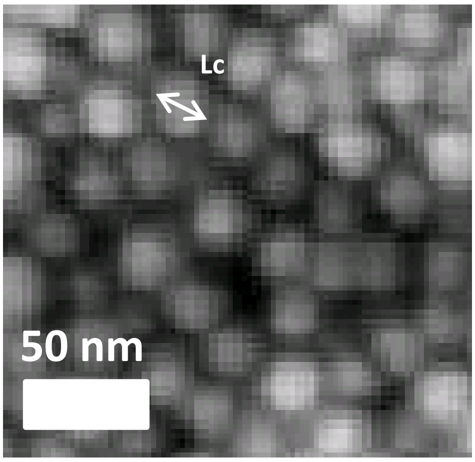

Figure S4. Illustration of the Lc on the AFM topography image. Lc represents the size of one spherical micelle.

Porosity of the film

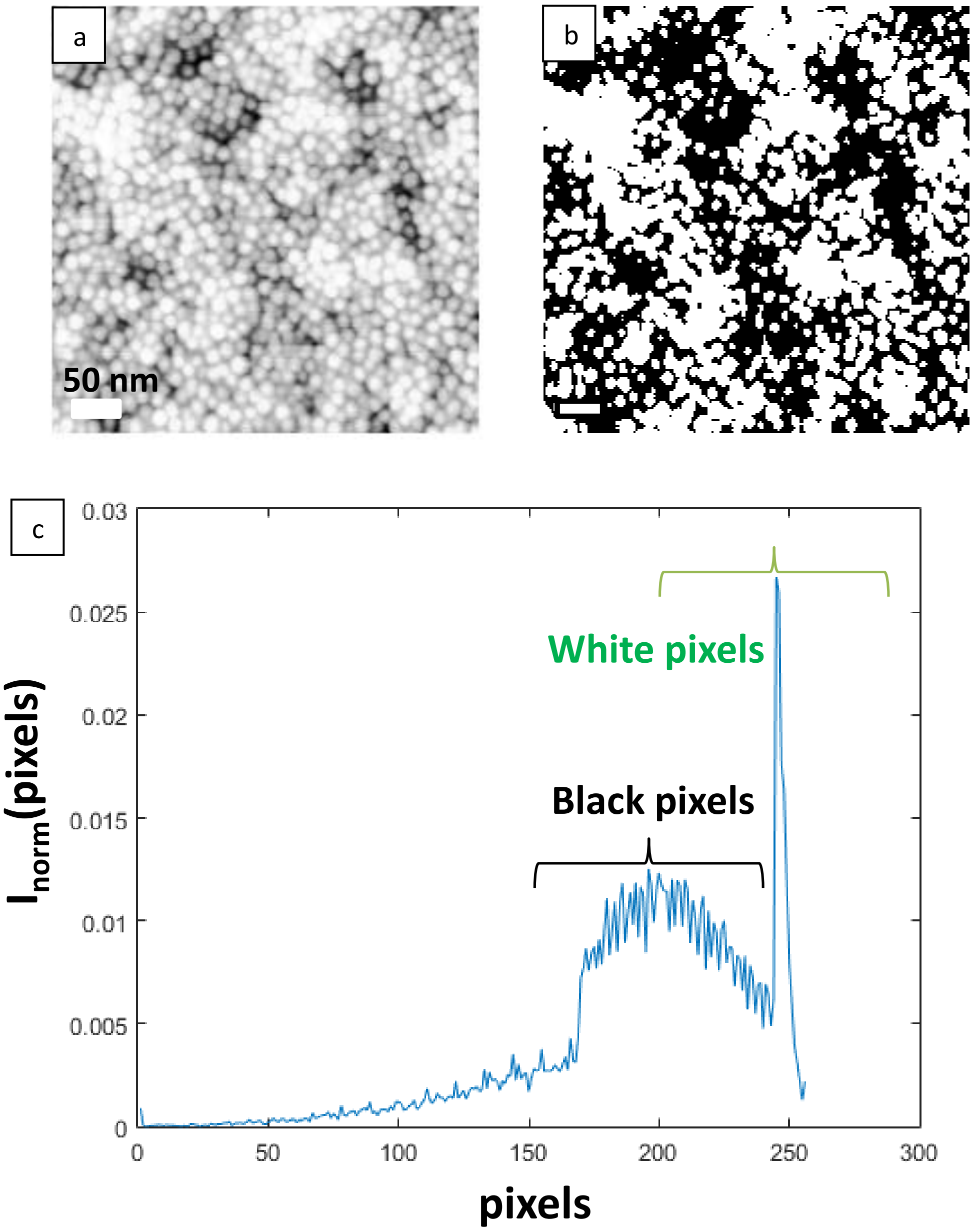

Figure S5. (a) SEM image of PS_{10K}-$PNaSS_{10K}$-PS_{10K} after drying by spin coating and detachment from the wafer by immersion in water (b) image (a) binarized using Matlab software (c) Diagram of Normalized intensity of the pixels versus pixel obtained by the data treatment on image (b)using Matlab software.

The porosity is calculated with the following Eq S2:

$$Porosity = \frac{Black_{\ pixel}}{Black_{pixels} + White_{pixels}} \times 100 \ (\ S2)$$

Filtration test supplementary information

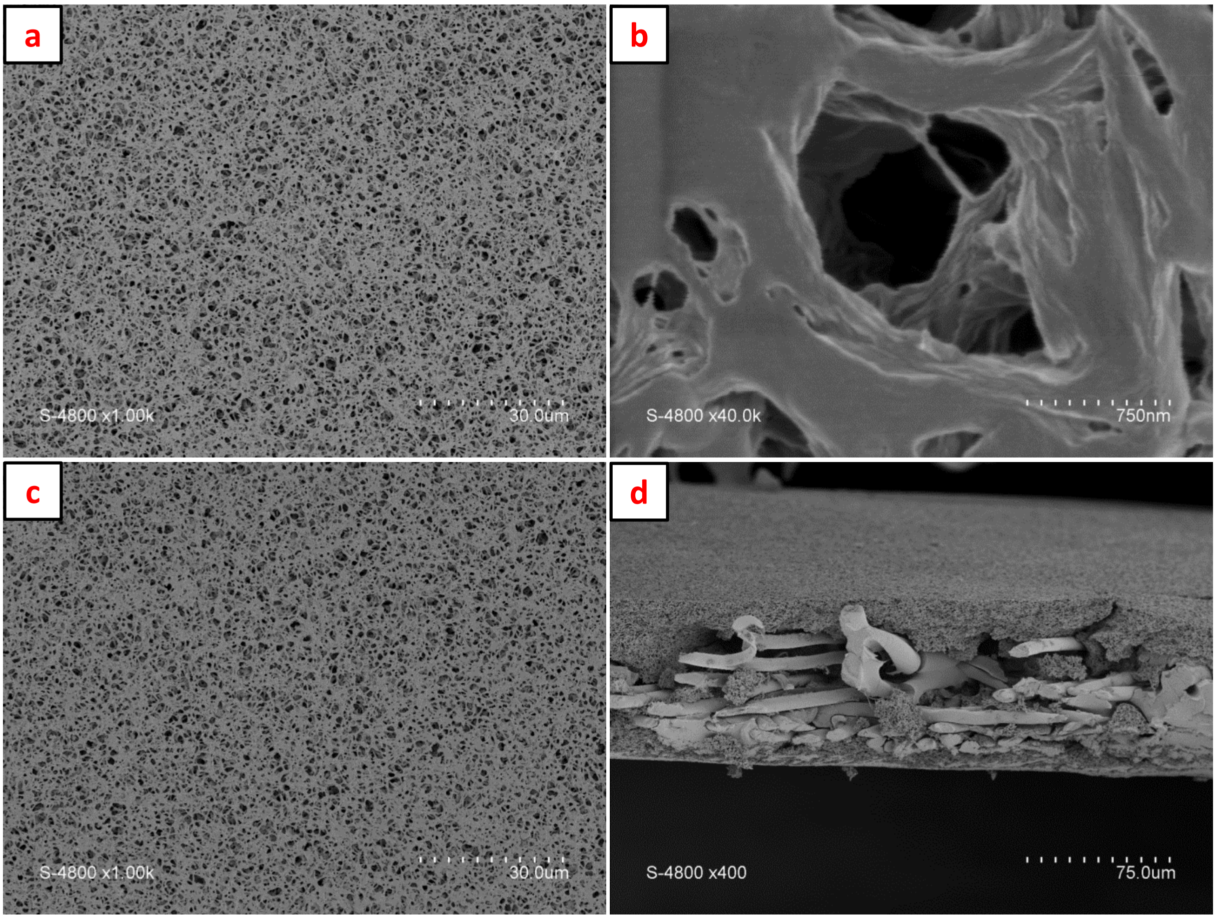

Figure S6. SEM images of the virgin Nylon membrane used as support for the coating of PS_{10K}-$PNaSS_{10K}$-PS_{10K} (a) Top surface (b) Zoom of the top surface (c) Bottom surface (d) Cross section of the uncoated membrane.

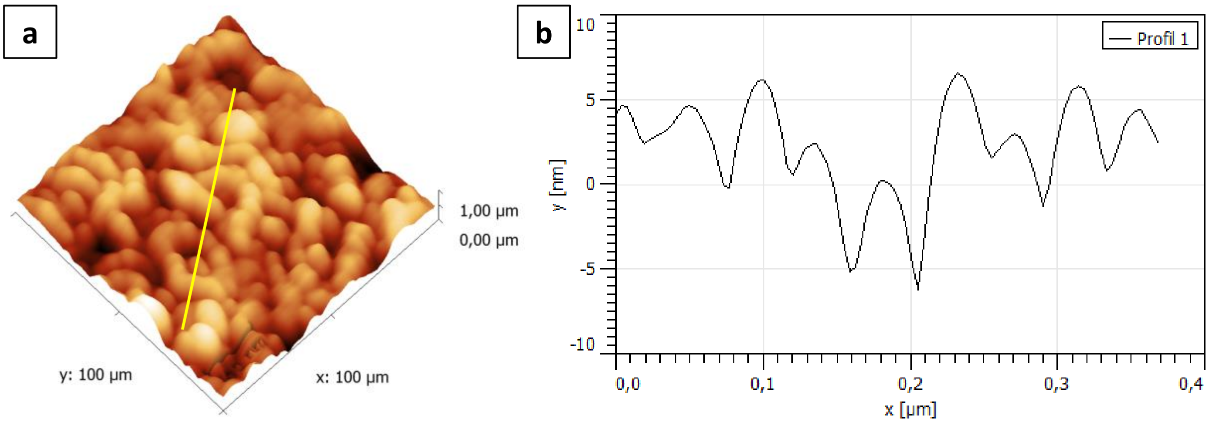

Figure S7. AFM 3D view and profile of the coated membrane after filtration cycles under pressure.

Zeta potential measurements

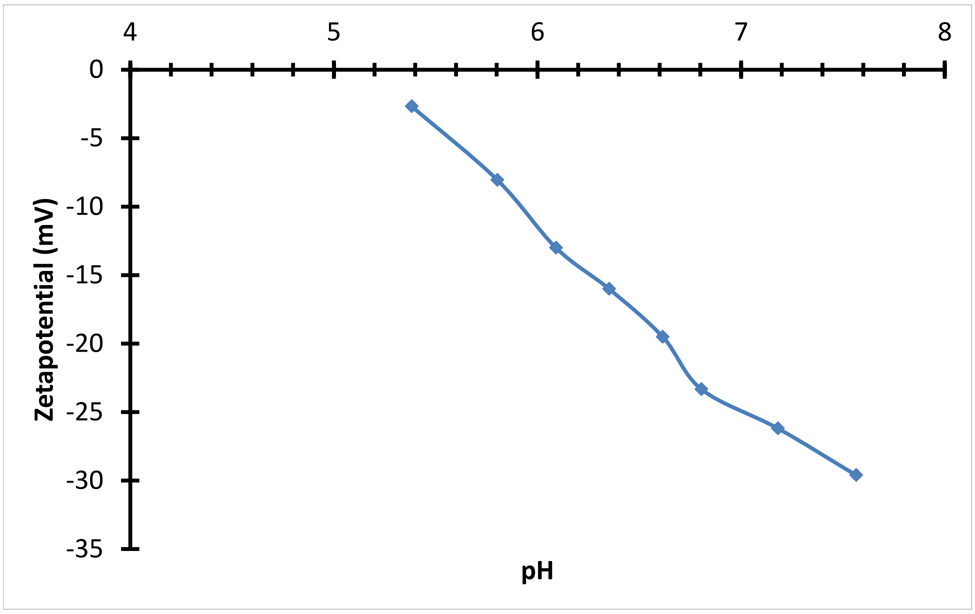

Figure S8. Zeta potential curve versus pH for the uncoated commercial Nylon membrane

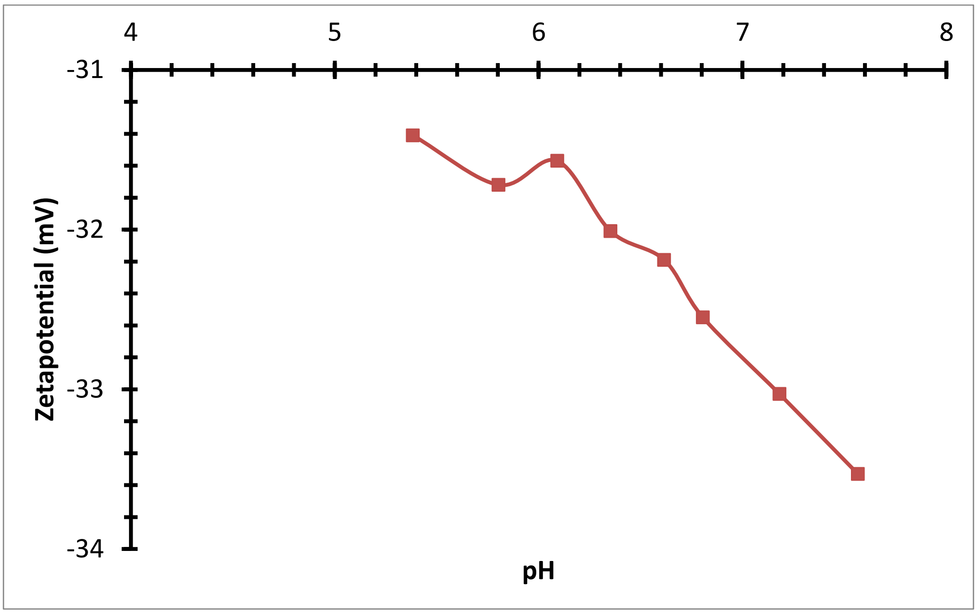

Figure S9. Zeta potential curve versus pH for the coated commercial Nylon membrane with flower-like micelles of PS_{10K}-$PNaSS_{10K}$-PS_{10K}
